# *‘The stars seem aligned’:* a qualitative study to understand the effects of context on scale-up of maternal and newborn health innovations in Ethiopia, India and Nigeria

**DOI:** 10.1186/s12992-016-0218-0

**Published:** 2016-11-25

**Authors:** Neil Spicer, Della Berhanu, Dipankar Bhattacharya, Ritgak Dimka Tilley-Gyado, Meenakshi Gautham, Joanna Schellenberg, Addis Tamire-Woldemariam, Nasir Umar, Deepthi Wickremasinghe

**Affiliations:** 1 London School of Hygiene & Tropical Medicine, 15-17 Tavistock Place, London, UK; 2 Save the Children, 1st & 2nd Floor, Plot No. 18, Sector - 44, Gurgaon, Haryana 122003 India; 3 Health Hub, The Office, 4th Floor, Ropp House, Adetokunbo Ademola Crescent, Wuse II, Abuja, Nigeria; 4 Jarco Consulting, PO Box 43107, Gofa Sefer, Addis Ababa Ethiopia; 5 Federal Ministry of Health of Ethiopia, PO Box 1234, Addis Ababa, Ethiopia

**Keywords:** Uttar Pradesh, India, Northeast Nigeria, Ethiopia, Scale-up, Maternal and newborn health, Innovations, Context, Health policy and systems

## Abstract

**Background:**

Donors commonly fund innovative interventions to improve health in the hope that governments of low and middle-income countries will scale-up those that are shown to be effective. Yet innovations can be slow to be adopted by country governments and implemented at scale. Our study explores this problem by identifying key contextual factors influencing scale-up of maternal and newborn health innovations in three low-income settings: Ethiopia, the six states of northeast Nigeria and Uttar Pradesh state in India.

**Methods:**

We conducted 150 semi-structured interviews in 2012/13 with stakeholders from government, development partner agencies, externally funded implementers including civil society organisations, academic institutions and professional associations to understand scale-up of innovations to improve the health of mothers and newborns these study settings. We analysed interview data with the aid of a common analytic framework to enable cross-country comparison, with Nvivo to code themes.

**Results:**

We found that multiple contextual factors enabled and undermined attempts to catalyse scale-up of donor-funded maternal and newborn health innovations. Factors influencing government decisions to accept innovations at scale included: how health policy decisions are made; prioritising and funding maternal and newborn health; and development partner harmonisation. Factors influencing the implementation of innovations at scale included: health systems capacity in the three settings; and security in northeast Nigeria. Contextual factors influencing beneficiary communities’ uptake of innovations at scale included: sociocultural contexts; and access to healthcare.

**Conclusions:**

We conclude that context is critical: externally funded implementers need to assess and adapt for contexts if they are to successfully position an innovation for scale-up.

## Background

In the Sustainable Development Goal era there remains strong interest in developing innovative health interventions to improve the health of populations in low- and middle-income countries. Donors commonly introduce comparatively small-scale, time-limited innovations that aim to improve health and are new to a particular context in anticipation that host governments will finance and implement at scale those that are shown to be effective. Yet these interventions commonly end when donor funding ends, which seriously undermines the value of externally-funded health programmes ([11, 16, 21]). In this paper we examine why interventions that improve health outcomes are rarely scaled-up by assessing the contextual factors acting as barriers and enablers in three low-income settings.

‘Scale-up’ has been defined in different ways including increasing the geographical reach of a programme for greater numbers of people and increasing financial, capital and human inputs to achieve this (Mangham and Hanson [10]). Following Mangham and Hanson ([10]) our working definition of scale-up is: ‘…*an increase in the coverage of health interventions that have been tested in pilot and experimental projects in order to benefit more people*…’. There is an extensive literature describing the barriers and enablers to scaling-up innovative technologies and practices. Factors include an innovation’s attributes such as its simplicity, relative advantage and adaptability. The needs, attitudes, knowledge and skills of potential adopters affect their acceptance of innovations, while opinion leaders and policy champions can influence government, health workers and communities to adopt innovations [2–4, 9, 12, 15, 17–20]. Country contexts can also influence scale-up. Policy making contexts include political regimes and ideologies, systems of governance, accountability and bureaucracy, ways policy ideas are understood and presented and donors, nongovernmental organisations and other policy actors’ influence on policy priorities [1, 5, 7, 14]. A country’s economic context includes the distribution of financial and other resources, macroeconomic policies, economic growth, inflation and debt [1, 7]. Health systems contexts comprise sector politics and priorities, human resources, infrastructure and commodity supply systems, health system management, finances and financing mechanisms [1, 5]. Socioeconomic and cultural contextual factors include gender relations and other social hierarchies, religious institutions and ideas, access to housing, employment and education [1, 8, 14].

We conducted a multi-country qualitative study to improve understanding of the contextual factors influencing government scale-up of externally funded maternal and newborn health (MNH) innovations that are shown to be effective and delivered to mothers and newborns in rural areas of Ethiopia, Uttar Pradesh in India and the six states of northeast Nigeria. As an operational definition of scale-up we assumed an innovation had been scaled if:

- Government had adopted the innovation as part of an existing or new government-led health programme;
- Government had agreed to finance the innovation’s implementation after external funding had ended;
- The innovation’s geographical reach had increased beyond externally funded implementers’ programme districts to benefit a greater number of people.

For our study, our focus was with how relatively small scale innovations are developed, delivered, evaluated and positioned for scale-up by implementers funded by bilateral donors and philanthropic foundations, and understanding the contextual factors influencing scale-up. These innovations aim to improve existing approaches, or introduce new ones, often strengthening government MNH services in rural settings as follows:

- Develop capacity of frontline workers including traditional birth attendants and community health workers, broaden their roles and introduce incentives, to improve service delivery
- Introduce tools, including communication aids, mobile phone technologies and quality assurance tools, to enhance frontline workers’ performance
- Strengthen healthcare referral systems, including emergency transport schemes, call centres and strengthening health workers’ capacities to make referrals in order to increase facility deliveries
- Strengthen community structures, encouraging behaviour change and local decision making to increase demand for services

## Methods

We adopted a health policy analysis approach informed by the stages heuristic framework [13] that identifies sequential stages in the policy process: agenda setting, policy formulation and policy implementation, and on the literature on scale-up and context to frame different contextual domains. From this we developed a framework to guide our study consisting of three distinct stages that are critical to scale-up:

- contextual factors influencing government decisions to accept, adopt and finance health innovations at scale;
- contextual factors influencing the implementation of innovations at scale;
- contextual factors influencing the willingness and ability of communities to accept and take up innovations at scale.

Having used these categories to develop a topic guide, researchers from Nigeria, Ethiopia, India and the UK piloted it at a workshop in Addis Ababa leading to minor adaptations being made to reflect different country contexts. Researchers used the guide to conduct semi-structured interviews with purposively selected stakeholders working in MNH, or having substantial experience and/or knowledge of issues relating to scale-up of MNH innovations including policy, financing and health systems issues. The interviewees were drawn from different sectors: government, development partners, civil society organisations (CSOs) including implementers of donor- funded MNH programmes, academic institutions and professional associations. Interviewees were managers and directors, programme officers and research and evaluation and technical officers. Fifty interviews were conducted in each of the three settings between July 2012 and April 2013.

Our sample of interviewees represent the majority of implementer and development partner organisations working on MNH in each of our three settings. We have deliberately not named specific organisations in our paper because of our commitment to maintaining respondent confidentiality. The MNH implementers we sampled are characterised as follows: the majority were large international nongovernmental organisations or large local nongovernmental organisations, together with a smaller number of US-based universities and for-profit consultancy companies implementing MNH programmes. Most of these implementers had in the past received large grants from different donors to maintain particular interventions and some were receiving multiple grants for separate pieces of work at the time of the interviews. Many implementers also worked with smaller local CSOs to implement work packages in particular locations. While a substantial amount of externally funded MNH-related work in the three settings took the form of projects to develop innovative interventions, some implementers also received donor funding for direct technical support to government agencies as well as advocacy work. The development partners we sampled included a mix of donors - bilateral agencies and philanthropic foundations - and UN agencies, some of which also funded MNH innovations. In addition to funding projects some development partners also contributed to larger health programmes, provided technical support for government, and in Ethiopia contributed to a pooled fund for work corresponding to the Millennium Development Goals. The MNH projects we explored in our interviews generally lasted up to five years and more commonly three to four years. The scale varied from a small handful of districts to several districts across multiple states or regions, and some were part of large multi-country grants. Some projects involved single innovations, while others involved a package of connected innovations.

The interviewers included NS, RDTG, DBh and ATW, and other researchers with training in qualitative methods. The interviews were conducted in private spaces to preserve confidentiality and all respondents gave informed consent before the interview. Where it was agreed with the respondent, a sound recorder was used for data capture. Interviewers wrote ‘expanded field notes’ [6] shortly after the interview comprising detailed notes arranged under thematic headings, with direct quotes to illustrate respondents’ voices. Through simultaneously capturing and analysing data, interviewers identified emerging interpretations and hypotheses to explore in ensuing interviews.

We adopted several steps to maximise the validity of our findings. We adopted an investigator triangulation approach to compare and agree researchers’ interpretations; this helped reinforce the validity of the results reported because each set of expanded field notes was the work of multiple researchers. Moreover, an analysis workshop enabled us to reach consensus on interpretations among researchers involved in the study and cross-country comparisons. Our relatively large qualitative sample, with interviewees from a variety of organisations, helped balance the views we present, and cross-checks of interviewees’ views enabled us to triangulate findings. We also conducted member checks: we presented emerging findings to interviewees and other relevant country stakeholders in Addis Ababa, Abuja and Lucknow who were invited to comment on the accuracy of our messages.

The analysis of the interview data was undertaken in five stages: 1) an analysis workshop in London at which NS, DW ATW, RD and DBh reviewed and agreed emerging findings and developed an analytic framework to enable us to directly compare our three study settings; 2) using Nvivo Version 10, NS and DW analysed the expanded field notes, using a framework approach to code *a priori* and emerging themes; 3) the analytic framework was used to organise the emerging themes ; 4) NS drafted the paper, which was then reviewed by all authors to ensure that the findings are represented coherently and accurately.

In order to maintain anonymity of our interviewees it is not appropriate to make the qualitative dataset supporting the conclusions of this article publically available.

## Results

A number of implementers we interviewed reported that the innovations they had developed had been scaled by government – or elements of an innovation had been adopted within government practices, although most were actively attempting to position their work for scale-up at the time of the interviews. Nevertheless, implementers emphasised that they had experienced many challenges to scaling innovations within the contexts in which they were working. Based on our framework and data from our 150 interviews, the key contextual factors that influenced attempts by externally funded implementers to catalyse scale-up of their MNH innovations are summarised in Table 1 below. The following sections present each of these factors in detail.

**Table 1:** Analytic framework: contextual barriers and enablers to scale-up

	CONTEXTUAL BARRIERS AND ENABLERS
Contextual factors influencing government decisions to accept, adopt and finance innovations at scale	How health policies are made • Government willingness to collaborate with development partners and implementers • Government responsiveness to civil society • Evidence-based decision making • Turnaround of government officials • Bureaucratic government institutions Prioritising and funding maternal and newborn health • National policy frameworks • Economic resources and global and development partners’ influence • Influence of powerful country actors Development partner harmonisation • Information sharing and coordinated communication with government • Embracing donor coordination mechanisms
Contextual factors influencing the implementation of innovations at scale	Health systems capacity • Health infrastructure • Human resources • Logistics and commodity supply • Health systems governance and health information systems • Financing Security context
Contextual factors influencing community willingness and ability to accept and take up innovations at scale	Sociocultural contexts and demand for healthcare • Education and awareness of health issues • ‘Traditional’ health-related beliefs and practices • Hegemonic gender relations • Heterogeneity Access to healthcare • Geographical distances • Poverty

## Contextual factors influencing government decisions to accept, adopt and finance innovations at scale

### How health policy decisions are made: *‘people are beginning to make demands on government’*

Our interviewees identified a number of aspects of the ways health policy decisions were made that influenced scale-up in the three settings: government willingness to collaborate with development partners and their implementers; government responsiveness to civil society; whether health policy decisions were based on evidence; turnaround of government officials; and bureaucratic government institutions.

#### Government willingness to collaborate with development partners and implementers

Interviewees suggested the governments in the three settings were open in principle to collaborating with development partners and implementers, and hence responded positively to innovations that align with their national plans, priorities and political thinking. Interviewees observed the Ethiopian government’s willingness to work with development partners and implementers that supported its aims within health sector programmes: *‘…the government has keen interest to work with any partners and to collaborate with them…to improve MNH in the country…’* said one, from a civil society organisation. Similarly, many northeast Nigerian states welcomed external partners bringing funding for MNH and other health programmes; Gombe, for example, was described as having an ‘*open door policy’* to such programmes. Interviewees expressed high expectations about the Uttar Pradesh state administration that came into power in 2012, with the young and energetic new state First Minister’s openness to new ideas and working with development partners and their implementers*.* Despite these signs, the state government was said to be living in the shadow of the prior regime which was less open to collaboration: *‘…the government sector is still paralysed with apathy, lethargy, lack of ideas…’* said an interviewee from a civil society organisation.

#### Government responsiveness to civil society

According to our interviewees, government responsiveness to civil society also influenced externally funded implementers’ efforts to catalyse innovation scale-up since most implementers were civil society organisations, and across the three settings the situation was changing. The Uttar Pradesh state administration was responsive to civil society, which, an academic interviewee felt was beginning to be viewed as a *‘force of change’*. Interviewees in Nigeria noted that stronger democracy meant an increasingly active civil society had influenced the allocation of resources; for example, CSOs advocated successfully for free maternal and child healthcare, leading to a bill being passed: ‘*As democracy becomes entrenched, people are beginning to make demands on government and as people make demands, government wants to show results…’* said an interviewee from a multilateral organisation. In both Nigeria and Uttar Pradesh our respondents pointed to organised networks of CSOs working together to influence policy decisions and in many cases they had been successful. In Ethiopia where civil society was less established, CSOs were also described as having some influence on government; one interviewee suggested: *‘civil society organisations can show strategic directions to policy implementation…they can also convince [the Ministry of Health] with evidence about their innovations to be taken up and delivered at scale…’.*

#### Evidence-based decision making

Our respondents suggested that the extent to which governments based policy decisions on evidence was an important consideration for scale-up. In practice when externally funded implementers had presented evidence demonstrating the effectiveness of their innovations this had tended to have limited influence on government thinking. A Nigerian interviewee in national government captured the problem of politics shaping decision making: ‘*The ideal situation is that evidence from research, pilots, or practice should influence government’s decision to shape policy. [But] in a country like Nigeria…people want to score cheap goals for political reasons’.* Similarly, in Uttar Pradesh, a civil society interviewee reflected: *‘…policies aren’t always based on evidence - sometimes huge decisions are made within an hour!’* In all three settings, ministers of health and state chief ministers tended to dominate decisions: ‘*whims of the power centres*’ as an academic interviewee in India put it*.* Nigerian state governors and other high ranking officials were reported as being motivated by ‘political capital’, as a national government interviewee reflected: ‘*When an individual is appointed or elected into political offices his associates see it as an opportunity to influence things and get favours. And because he wants to please his ring of friends and associates that makes decision making not quite representative’.*

#### Turnaround of government officials

Turnaround of officials at all levels undermined efforts to catalyse scale-up as reshuffling and attrition was constant across the three settings - *‘fickleness in the entire system’* as an Indian civil society interviewee observed. This made securing government agreement transitory; new officials were often unwilling to accept their predecessors’ decisions to scale-up innovations. One key informant noted: *‘…once your idea has got the desired approval the person may have changed’.* In Uttar Pradesh and Ethiopia our interviewees reflected on the limited time a new leader or official had in which to learn their job. Poor institutional memory retained by the system when individuals leave, and outgoing parties’ unwillingness to share knowledge with new administrations, were related factors.

#### Bureaucratic government institutions

Bureaucratic institutions were also reported as a barrier to scale-up. Complex, lengthy government approval processes undermined or delayed decision making and slowed or stalled the implementation of innovations at scale. A key informant in India observed: *‘Right from the NRHM [National Rural Health Mission] directorate to the planning commission there are tedious processes to get approvals, once approvals are made there are bureaucratic delays…’.* Indeed, some procedures became progressively complex; a corruption scandal surrounding the use of NRHM (now known as the National Health Mission) monies prompted the incoming Uttar Pradesh Government to route financing through the Treasury to strengthen checks and balances. Ethiopian procurement and contracting rules were also depicted as constraining the adoption of new commodities or innovative practices: *‘They can’t do things in certain ways because the government rules are very rigid and constraining…*’ said a representative of a donor agency.

### Prioritising and funding maternal and newborn health: *‘the stars seem aligned’*

Interviewees suggested that the willingness and ability of governments in the three contexts to scale MNH innovations closely reflected the prioritisation of MNH in federal and state policies. Our data reveal a number of factors connected to policy prioritisation: the existence of national policy frameworks; the availability of economic resources; global and development partners’ influence; and the influence of professional associations, traditional leaders and media.

#### National policy frameworks

The high priority given to MNH in Uttar Pradesh and Ethiopia was enshrined in policy frameworks which our interviewees described as enabling MNH innovation scale-up. The federal government of India’s NRHM was a positive policy environment bringing with it substantial funding for state governments’ rural primary healthcare programmes, including MNH programmes. The new Uttar Pradesh state administration in combination with the NRHM was seen by interviewees as an important policy window for externally funded implementers to put forward innovations that align with the state’s aims: *‘The stars seem aligned in terms of the [policy] environment!’* one interviewee from a donor organisation exclaimed, while another, from a multilateral agency, reflected: ‘*It’s important that institutions capitalise on this mood’.* Ethiopia’s prioritisation of rural primary healthcare was embodied in its national flagship programme – the *Health Extension Program* (HEP) and national health plans including the Health Sector Development Program IV 2010/11-2014/15. The Ethiopian Government was described as receptive to externally funded MNH interventions that align closely with national plans and priorities, as a civil society organisation representative explained: ‘…*government policies and programmes are very supportive to our programme…this is an encouraging issue for this organisation to expand its interventions…’*. In contrast, rural primary healthcare, including MNH, struggled for policy attention in Nigeria: ‘*What’s now happening is there’s erosion of primary healthcare,’* said a state government interviewee. The problem stemmed less from Nigerian economic resources, and more from how resources were allocated. Health was not on the executive list in the 1999 Constitution; at the time of the interviews it was not considered a priority sector and competed annually for funding: ‘*There’s a lot of politicking and jostling for a piece of the cake…you struggle for monies to come to maternal and newborn health,*’ a researcher said. State health departments therefore had limited finances to divert to scaling external programmes.

#### Economic resources and global and development partners’ influence

India’s economic growth together with the NRHM substantially increased Uttar Pradesh’s resources for rural healthcare including MNH. Reductions in external aid receipts changed relationships between donors and the state government, with the former increasingly adopting technical assistance rather than funding roles which gave them less influence on state policies, and made it crucial for externally funded programmes to closely align with Uttar Pradesh’s priorities. A government interviewee suggested: *‘…ideas that are working within the government framework have greater potential to be scaled-up. Working in oblivion doesn’t help…’.* In Ethiopia, domestic resources were more limited: ‘*A big barrier to scale is resources and continuity of resources’* as one donor representative noted. While the Ethiopian Government maintained strong control over its policy priorities, substantial external aid was required to support its health programmes and global priorities such as the Millennium Development Goals (MDGs) had shaped Ethiopia’s health programmes. Interviewees also reported that the publication of the 2011 Demographic and Health Survey revealed disappointing improvements in neonatal and maternal mortality against MDG targets which reinvigorated the Government’s efforts in MNH, and externally funded implementers saw this as an opportunity to promote their MNH innovations. An interviewee from the government observed that at the time of the interviews: *‘We are still lagging behind the MDG targets…there will be no change in priority until the MDGs are met in the coming three years - MNH will continue to be our top priority…’.* In northeast Nigeria, while state governments commonly support programmes in principle, they were not always backed by financial resources: ‘…*a lot of rhetoric – they don’t put their money where their mouth is…,*’ noted an academic researcher. One reason for state governments’ limited financial support for rural healthcare was donor attention on this issue: *‘everything is seen as if it has to be donor-funded,’* said a multilateral agency representative. As a consequence, resources were vulnerable to shifting global priorities; HIV, for example, competed with MNH for funding and attention.

#### Influence of powerful country actors

Powerful actors also influenced the introduction of certain MNH interventions in Nigeria. Professional medical associations opposed community health workers dispensing the drug Misoprostol to prevent and treat postpartum haemorrhage: *‘…they have knowledge, power, they think they know what to do…so relinquishing power was a major problem for them,*’ said one academic researcher. While traditional rulers had no formal role in government decision making in reality their influence was substantial. Individual rulers often resisted - although sometimes supported - ‘western’ health interventions making it difficult to introduce them in some states. Family planning, which was often conflated with MNH, was particularly controversial since many people believe it contradicts Islamic teaching and hence traditional leaders can oppose it. These problems appear to be intensifying, as an interviewee from a donor organisation clarified: *‘…all the social pressure at this point is to regress to a more conservative, historical set of behaviours. Everything we are talking about involves some degree of modernisation and the cultural current is absolutely against that at this point’.* Nevertheless, interviewees noted changes in federal government’s commitment to MNH in the form of new funds, and some state governments introduced free MNH services. One reason was the government’s attitude towards evidence, coupled with strong civil society advocacy and greater media attention on maternal and child mortality-related issues. A 2008 report presented at a public meeting highlighted high maternal mortality rates in Nigeria which attracted officials’ attention, and data on Gombe pressed the state government into acting. As one national government representative remarked: ‘*Any responsive government will respond to such pressure to look responsible’*.

### Development partner harmonisation: ‘*government is very good at Balkanising us’*

An important barrier to scale-up emerging from our study was poor harmonisation among the many donors and other development partners and externally funded implementers including the multiplicity of smaller local CSOs implementing parts of wider programmes in the three settings. Harmonisation was made difficult by competing interests, priorities and mandates and pressure to attribute outcomes to specific donor funding inputs. Competition among implementers for donor funding with the expectation that they deliver results to ambitious timeframes thwarted programmatic coordination and information sharing. Implementers feared their ideas for innovations would be poached jeopardising their competitive advantage among rivals: ‘*…the issue of competition is crazy!’* exclaimed an interviewee from a Nigerian civil society organisation.

#### Information sharing and coordinated communication with government

Poor harmonisation undermined scale-up in different ways. It weakened government strategic oversight of external programmes making it difficult to coordinate and deploy externally funded innovations at scale resulting in duplication and programmatic gaps. Interviewees described limited information sharing as a missed opportunity to strengthen innovation design by building on learning derived from programmatic experiences, as a civil society interviewee captured: *‘People in India are not combining their expertise…instead of wasting time reinventing the wheel we really need everyone to come together…’.* Further, donors and implementers competing for attention made it difficult for government to make informed decisions about scaling-up: *‘…it’s our moral and ethical duty to work together…we have to go beyond our little thing and make sure that we’re asking for common asks that are based on evidence…’* a civil society interviewee from India suggested.

#### Embracing donor coordination mechanisms

Many interviewees agreed these problems could be mitigated through donors and implementers working through government-led partner coordination mechanisms, including the *Technical Working Group* in Ethiopia, the *Health Partners’ Forum* in Uttar Pradesh and Nigeria’s *Maternal and Newborn Child Health Core Technical Committee.* In Ethiopia interviewees were most positive about their government’s efforts to coordinate donor programmes, with the Technical Working Group emerging as an important vehicle for achieving this: ‘*The government is very good at Balkanising [separating] us – there is very little overlap*…’ according to one interviewee from a donor agency, while a government interviewee said: *‘All plans are discussed with partners and we put together an action plan - all the bad and good experiences are discussed…’.* An interviewee from a multilateral development agency in India, however, complained about limited donor engagement in the Uttar Pradesh mechanism: *‘Though this Health Partners’ Forum has potential it’s underutilised…’.* The Nigerian government and key development partners, responding to the International Health Partnership, signed a Compact on Health in 2011 which strengthened commitments to harmonising health programmes under the National Strategic Health Plan. Interviewees reported that this had stimulated better coordination and represented a more conducive environment for scale-up: *‘Donors have a forum where they meet regularly and integration among donors has improved over the years…but there’s still a lot to be done…,’* said one, from a civil society organisation.

## Contextual factors influencing the implementation of innovations at scale

### Health systems capacity: *‘They can be burned out easily*’

The capacity of the government health systems in the three settings was a critical barrier to scale-up; interviewees explained that it was difficult to ‘layer’ innovations onto chronically weak systems, as a development partner lamented about the situation in India: *‘…we try to scale-up things through a broken system - it’s difficult to succeed in that context’.* Similarly, a development partner in Nigeria said: ‘*…there are so many gaps in the system…there are too many areas you need to fix’.* Key factors influencing scale-up emerging from our interviews are described below framed using the World Health Organisation’s Six Building Blocks of health infrastructure, human resources, commodity supply, health systems governance, health information systems and health systems financing.

#### Health infrastructure

Our interviewees cited health infrastructure as an important barrier to scaling innovations. Rural health posts were depicted as very basic: they lacked water and electricity, sanitation and telephones, and were crumbling and unhygienic, which made it difficult to prevent infection. According to an interviewee from a multilateral development agency in Ethiopia: ‘*Imagine a health centre without water, sanitation and power for pregnant mothers to come and deliver in…’.* Interviewees commented that it is difficult to scale-up innovations through the lowest level rural health clinics: *‘In smaller health clinics the conditions are so bad this project may not in fact work very well…until the supply side is straightened out I think [this project] is bound to fail,’* said one implementation grantee in India. Indeed, poor services undermined confidence among potential beneficiaries – representing a further barrier to scaling facility-based innovations. An Ethiopian government interviewee said: *‘An unsatisfied client is unlikely to come back…’,* while a donor said of Nigeria: ‘…*when you create demand and there’s no supply then you have people who are disillusioned - people who feel betrayed are not willing to access the system anymore’.*

#### Human resources

Government efforts to expand the health workforce by recruiting and training community level health workers were significant, namely Accredited Social Health Activists (ASHAs) in India, Health Extension Workers (HEWs) in Ethiopia and Junior Community Health Extension Workers and Community Health Extension Workers in Nigeria. Despite this, at rural primary healthcare level in the three settings, there remained staff shortages, poor training, problems of staff attitudes, high workloads and unsatisfactory incentive systems. Limited girls’ schooling in northern Nigeria and high illiteracy among women in Uttar Pradesh were reported as underlying shortages of women health workers in those settings, which an interviewee from an implementing grantee explained was a serious problem: ‘*Sometimes women don’t want to go to a delivery facility because there are only men there…’.* This was a critical barrier to scale-up, as many of the MNH innovations described by interviewees related to strengthening existing healthcare workers’ capacities and expanding their roles: *‘…the whole system’s a shambles – how do you scale-up without people?’* asked an academic in India.

Interviewees reported that health workers in the three settings were not unwilling to accept innovations, provided they helped them to achieve their tasks and did not place an additional burden on them. Nevertheless, health workers’ attitudes had been a barrier to scaling community-based innovations. An interviewee in Nigeria recalled health workers speaking to patients rudely, disregarding their fears and preferences and withholding care, while an interviewee from a multilateral agency in Ethiopia echoed: *‘If I come with my labouring wife at midnight to a health facility and he/she says “no, we are asleep, come in the morning”, why do I come to him/her again?’.* In Nigeria and Uttar Pradesh community health workers were often recruited through family connections rather than based on qualifications or ability. ASHAs were selected through the *panchayat* (local self-government) system: *‘…most [frontline health workers] belong to the family of influential people in the village – or as we say the dominant castes’,* which influences attitudes to low caste families: *‘Contamination of their caste system virtues, mixing up with other castes [is a problem for them],’* said an implementation grantee. High workloads and unsatisfactory incentive systems reinforced these problems. Workloads increased as each additional donor programme added new tasks and required new procedures: *‘Every new programme…you have a new set of forms…that kinda adds a lot of workload…’* explained an implementation grantee in India. In Nigeria a civil society interviewee said: *‘You see one health worker conducting twenty to thirty deliveries a day - it’s too much for her!’.* Workloads for Ethiopian HEWs posed a problem as they received a limited stipend and inevitably juggled their roles with economic and household activities, especially during peak agricultural seasons: ‘*They can be burned out easily*’ observed a key informant. Poor incentives in northeast Nigeria were linked to rural health worker retention: *‘…retention becomes a problem - they’ll gravitate to the city where they have money and access to services…,’* said a civil society interviewee. In Ethiopia, health staff receiving training through donor programmes commonly used this to seek better paid posts in urban areas: *‘Training is not a solution to problems we have!’* said a civil society representative.

#### Logistics and commodity supply

Weak logistics and commodity management systems in the three settings resulted in uneven continuity of supplies of essential drugs, vaccines, delivery kits and other consumables in lower level rural clinics, which interviewees agreed represented substantial barriers to scaling-up innovations that depend on the distribution of commodities. A bilateral donor in Nigeria described the problems stemming from interrupted commodity supply: *‘You can get people excited about a commodity and they will be willing to use it, but if the chain of supply stops, you are really disrupting the process and creating more problems’*. Lack of equipment, such as refrigerators as well as training to use and maintain equipment were related problems. A researcher in India said: *‘The majority of equipment even if installed is either not working, or there’s a lack of skilled operators who can handle the equipment’.*

#### Health systems governance and information systems

Health systems governance and health information systems were also described by our interviewees as barriers to scaling MNH interventions. Fragile management and supervision systems existed both within health facilities and sub-national health departments. Poor access to and synthesis of information undermined local level managers’ decision making and eroded health workers’ motivation to record activity data, as an interviewee from a multilateral agency in India explained: *‘It’s all one way traffic, collection of information happens, it’s fed in…it’s of no use to the supervisor who collects it’.* Limited technical capacity of managers undermined their ability to offer effective supportive supervision, and systems to ensure accountability for performance were weak: *‘No one is held accountable for maternal deaths,’* remarked a civil society representative in Nigeria. Weak monitoring systems were reported as undermining accountability and decision making, while limited training on data collection and recording led to inaccurate and missing data: *‘…we find funny figures! The figures are quite inconsistent…’* said a civil society interviewee in Ethiopia, while attempts to introduce electronic systems in the three settings were nascent and undermined by limited electricity supply and lack of computer training.

#### Financing

Inadequate funding was described by interviewees in the three settings as a problem underlying many health systems weaknesses: *‘I don’t think there’s any other barrier or constraint than funding,’* a civil society representative in Ethiopia observed. As discussed earlier, overall domestic resources were not necessarily the critical problem in India and Nigeria, and there were substantial donor contributions to support health programmes in Ethiopia: the problem stemmed from the allocation of resources across different sectors, and between urban and rural, tertiary, secondary and primary healthcare, and between MNH and other health priorities. Additionally, flows of finances through the system were commonly delayed or interrupted. The non-payment of healthcare workers’ salaries was cited by a donor interviewee as common in Nigeria: *‘If you look at the Federal budget in Nigeria there’s a lot of money that starts at the top of the system – that’s not a small budget. But most facilities see nothing. Many of them don’t have their staff salaries paid…’.* This inevitably undermined the motivation and therefore the retention of health workers.

### Security context in northeast Nigeria: *‘a total no-go area’*

A recurring issue in northeast Nigeria was the problem of security. Boko Haram opposed ‘western’ development programmes including efforts to change ‘traditional’ ideas and practices, which interviewees reported as a critical barrier to scaling MNH innovations, and indeed to delivering regular health services. Interruptions of services were frequent; it was difficult for staff to deliver both facility and home-based services, and for women to visit facilities during times of crisis. Curfew periods and harassment by security personnel were reported as disrupting service access as a donor explained: *‘Borno is a total no-go area…[it’s] almost continuously under curfew it’s almost impossible for mothers to get to health facilities…’.* It was also difficult to recruit and retain health staff and for international staff to travel to the northeast states, hence, some donors were cautious about embarking on new projects: ‘*I know of some organisations that just closed down their programmes in the north…,’* said a civil society interviewee.

## Contextual factors influencing community willingness and ability to accept and take up innovations at scale

### Sociocultural contexts and demand for healthcare: *‘demand creation is a serious challenge’*

Multiple socioeconomic and cultural factors influenced rural communities’ demand for scaled-up MNH innovations in the three settings.

#### Education and awareness of health issues

Respondents argued that communities’ limited awareness of health issues and services undermined their willingness to use MNH interventions and that illiteracy and low levels of education, especially among girls and women, underlie this. A civil society representative in India linked poverty and illiteracy with a lack of sense of entitlement: *‘…when we talk of maternal health we talk of women who are very poor, illiterate, who come from marginalised society. I think we fool ourselves, we’re romanticising, when we think those women are actually going to come out and ask for accountability’.* Nevertheless, some interviewees suggested communities rather than the health system were incorrectly blamed for problems of uptake: *‘No community is so dumb as to not understand its own benefits’* said a professional association interviewee in India.

#### Traditional health-related beliefs and practices

According to our interviewees ‘traditional’ health-related beliefs and practices among rural communities tended to be responsible for the slow adoption of new ideas: ‘…*traditional beliefs and some cultures are barriers to people not seeking care…demand creation is a serious challenge…,’* explained a civil society interviewee. A common discourse in rural Ethiopia was lack of control, with health being determined by god, illness being caused by the evil-eye and childbirth being constructed as a natural rather than medicalised event. Some communities were reported as not forming ties with newborns until a certain age due to high infant mortality*.* Reluctance to use facility-based services was observed by interviewees in Ethiopia and northeast Nigeria reflecting a preference for secluding newborns; women in northeast Nigeria were expected to demonstrate their strength by delivering at home, and birth-related ceremonies in Ethiopia reinforced the preference for home births, as a government interviewee explained: *‘Birth in this country is ceremonial. There’s a so-called porridge ceremony…they don’t want to miss that and health facilities can’t provide that’*.

#### Hegemonic gender relations

Hegemonic gender relations reinforced these issues; across the three settings men typically controlled household spending and health decision making, which was exacerbated by girls and women’s low education levels and early age of marriage, and in northeast Nigeria, polygamy. Such power relations were described by interviewees as highly engrained making change a slow process. A civil society representative in Nigeria explained: *‘…men see women as property; he dictates what needs to be done…’.* Hence, women needed their husbands’ permission before seeking medical attention outside their homes, and had to ask their husbands for money to do so. A civil society interviewee in Ethiopia said: *‘…males have a dominant role to decide on…household service seeking behaviour including MNH services…’.* Further, male healthcare providers, and indeed transport workers such as taxi drivers, were not readily accepted by many husbands. These factors represented major constraints to innovation scale-up, especially facility-based innovations.

#### Heterogeneity

Substantial heterogeneity was also reported within each of the three settings which made it difficult to scale-up innovations developed in one location to others without adaptation. Interviewees described local variations in religions, ethnicities and castes, climate, health problems, health-related behaviour and health systems capacity. The northeast states of Nigeria were depicted as very heterogeneous by a national government interviewee: *‘Every community is unique…even when they have the same structure they still have their own peculiarities’.* Similarly, in Ethiopia an interviewee from a multilateral agency explained: *‘…Ethiopia is a big geography…there are pastoralists, agrarians, and people living in different regions with different needs…so there’s no one single solution for all…’.*

### Access to healthcare: *‘poverty, poverty, poverty’*

There were multiple factors influencing healthcare access in the three settings.

#### Geographical distances

Interviewees cited geographical distance as a barrier to scale-up; low population densities across wide areas in Ethiopia and northeast Nigeria, especially arid areas inhabited by pastoralists, made uptake of interventions difficult, as a professional organisation interviewee explained: *‘…community groups who have access to roads, telecommunication and electric power usually accept and use innovations more than those who don’t…’.* Limited public and private transportation, poor roads, villages lacking vehicular access, difficult terrain and climate posed difficulties for some communities seeking healthcare and health workers reaching some communities. In Nigeria an implementation grantee explained: ‘*During the rainy season it’s very difficult to access some of the communities; it’s very dangerous, especially where there are no bridges’.* A common problem experienced by Ethiopian HEWs was their ability to travel to remote households and health posts on foot. Uttar Pradesh’s vast population - over two hundred million people - and geographical size posed particular barriers to scale-up as externally funded implementers typically operated within a handful of districts each representing two to three million people. An civil society interviewee summarised: *‘Uttar Pradesh has its own special set of problems – a huge population, I think huge percentage living under the poverty line…it’s huge…’.*

#### Poverty

Rural poverty and unemployment also inhibited scale-up. According to our interviewees, costs of transportation and receiving healthcare services could be prohibitive – despite MNH services being purported as free to users in the three settings: *‘Poverty, poverty, poverty!’* exclaimed an implementation grantee in Nigeria. An interviewee in India quoted a study that showed that despite institutional delivery being free, each woman paid on average an equivalent of US$200 in informal out-of-pocket costs. A Nigerian civil society organisation representative lamented: *‘Health expenditure per family is about 65% of their income…no wonder children are malnourished, no wonder nothing gets done, no wonder children aren’t going to school. They are spending more than half of what they earn on health - that’s not fair, that’s a big barrier’*. In Nigeria and Ethiopia rural communities’ incomes were also very seasonal; particular hardship during certain parts of the year made it difficult to seek healthcare.

## Discussion

Our study extends and deepens existing knowledge on innovation scale-up. Previous studies acknowledge that context influences scale-up (for example Hanson et al., 2003; [15, 17–19]). Our comparative study across three settings reinforces the importance of context: we conclude that donor agencies and their implementers cannot simply develop effective health innovations and generate robust evidence to demonstrate their impacts to guarantee scale-up. There are many contextual factors intervening which they need to respond to; achieving scale-up in these settings is very challenging and far from inevitable.

A contribution of our study is it usefully distinguishes between three distinct contextual domains that need to be taken into account when considering innovation scale-up: the contextual factors influencing government decisions to accept, adopt and finance at scale externally funded innovations; contextual factors influencing the implementation of an innovation at scale; and the contextual factors influencing whether communities are willing and able to access and take up innovations at scale. Based on these domains and on the qualitative data emerging from our study we believe our analytic framework (Table 1, above) will be useful for researchers studying the contextual barriers and enablers to scale-up and for donors and implementers planning health projects with scale-up in mind.

Existing literature tends to generalise the factors influencing scale-up across geographical settings or focuses on scale-up in a single country or region, whereas here, we draw out contextual differences and similarities across three diverse settings. Perhaps surprisingly, there were many aspects of the policy making context that were common across in the places we studied. Powerful government actors dominated decision making, often with limited reference to evidence. Government responsiveness and accountability to civil society was limited, although this was starting to change. Turnaround of officials undermined relationships and reversed decisions, while complex bureaucratic rules and procedures slowed or halted efforts to introduce innovations within government systems. An important factor that is not captured in the existing scale-up literature is limited harmonisation among development partners and their implementers, including lack of programmatic coordination and limited information sharing that affects government decisions relating to innovation scale-up, and more broadly to leadership and oversight over multiple donor-funded health programmes.

As well as similarities, there were key differences in the policy making contexts of the three settings. In Uttar Pradesh at the time of the interviews *‘the stars seem aligned’* with a combination of substantial funding through the NRHM and a state administration open to development partners and new ideas, although partners’ influence was circumscribed due to their decreasing contributions to health funding. Ethiopia retained tight control over health agendas, but unlike India, the country’s health programmes were dependent on donor funding. The government was, however, willing to rapidly adopt and scale-up innovations that it favoured – and like India had a flagship national programme for rural primary healthcare reflecting high levels of policy prioritisation for these issues. Hence, in both Uttar Pradesh and Ethiopia innovations aligned to key policy frameworks had a realistic prospect of being funded at scale by government, either alone or using donor funding. The same was not true among northeast Nigerian states where rural primary healthcare was constructed as a donor ambit and hence shortages of government health funding, coupled with a history of government reneging on commitments, meant that donors rather than state governments appeared to offer the best prospects of funding innovations at scale.

The three geographical settings had broadly similar problems of rural primary healthcare capacity that all represented barriers to scaling MNH innovations: crumbling infrastructure; shortages of trained health workers; weak logistics and commodity management systems, governance and monitoring systems. Externally funded implementers and donors were very conscious of the reality of trying to work *‘through a broken system’*. While the imperative to align with government policies, programmes and systems was acknowledged as essential to engender government acceptance, and potential funding for scale-up, the temptation to bypass government health systems in the interest of achieving expedient results within short project timeframes was not lost on donors and their implementers. A factor not reported the existing scale-up literature that was specific to northeast Nigeria was the security situation that undermined health worker retention and community access to health facilities. Coupled with resistance to ‘western’ health programmes from some religious leaders, this added to the challenges of scaling-up MNH innovations in that context. There were also multiple sociocultural, geographical and socioeconomic challenges to innovation uptake by beneficiary communities; as with most health systems issues we found these to be broadly similar across the three settings. An underlying factor was hegemonic gender relations closely connected to health beliefs and practices surrounding childbirth. Women’s isolation within the home, limited decision making power and control over household resources made facility-based innovations particularly challenging to scale-up.

There are several implications stemming from our research – specifically, several ways both externally funded implementers and their donors might take steps to respond to these contextual constraints. It is vital for implementers to position their innovations as both technically sound and closely aligned with national health priorities and programmes; to connect with and invoke the support of influential government actors; to respond to changing government administrations and officials including being prepared for repeated, continual advocacy; and involving government throughout the process is a critical underpinning of scale-up because this can engender government ownership of an innovation – which are factors raised elsewhere [2–4, 9, 15, 17–20]. Stepping up efforts at harmonisation is also important, including being prepared to share learning and insights, and coordinate communication to help governments make informed decisions about innovation scale-up. All of this requires building a systematic assessment stage into a grant to understand and anticipate the policy making context, policy priorities, government systems, institutions and procedures and the donor environment.

There are also ways externally funded implementers might act in response to the health systems barriers to scale-up reported in this paper. Acknowledging the problem of human resources is critical as most innovations we considered aim to work through existing health workers including strengthening their capacity and performance, hence innovations need to be designed to be easy to implement and beneficial to health workers so they are motivated to use them. Better programmatic harmonisation would also be beneficial in avoiding overburdening key community health workers and the health system in general. Again, an assessment stage within a grant would help anticipate and respond to health systems constraints including understanding the needs, priorities and attitudes of health workers. Designing innovations to be culturally acceptable in such contexts, including involving men as well as women in the design, is critical and might mitigate some of the multiple sociocultural problems pointed to earlier, as is ensuring there are clearly observable benefits to users. Heterogeneous socioeconomic, cultural and geographical contexts mean that designing an innovation that is easily adaptable to different contexts is also an important step. Building in a review period towards the end of a grant can also be valuable to help inform the modification of innovations for scale as well as drawing out learning about what worked well and why, and sharing that learning with government and other development partners.

Donors therefore need to encourage and enable their implementers to take these steps to respond to county contexts. They should allow implementers the flexibility to react to changing policy contexts such as unpredictable - yet inevitable - changes in country priorities and programmes that occur over time rather than insist on fixed project deliverables and timelines. Donors also need to find ways to encourage their implementers to share information about their project activities – as well as sharing information themselves on the programmes they support through government-led coordination mechanisms. They also need to avoid overly complex project monitoring and reporting requirements that burden health systems – not least health workers implementing externally funded innovations, and attempt to harmonise these with other donor funded health programmes to reduce the burden. Donors should be prepared to fund implementers to undertake systematic assessments of policy, health systems and sociocultural and geographical contexts to enable them to design their projects to be responsive to country contexts. This is likely to involve committing more resources over longer periods to enable their implementers to do so.

Our study has some limitations. We inevitably provide a simplified snap-shot of what are very complex, varied and changing country contexts, and were unable to measure the relative importance of the different contextual factors in those settings – although our data suggest all the issues we discuss are important. We elicited the experiences and views of decision makers’ but not those of health workers or communities who may have contrasting views to those we report in this paper. Our study focussed on specific, externally funded project-based innovations in the field of MNH. We did not explore broader health systems strengthening work related to MNH, nor to donors’ contributions to pooled funding in Ethiopia that may have been used for scaling MNH services. Additional research would be of great value to understand issues of innovation scale-up from health worker and beneficiaries’ perspectives and to compare the three settings in which our study was conducted with other locations.

## Conclusions

A key message from this paper is that externally funded implementers need to be conscious of and responsive to contexts if they are to successfully position a health innovation for scale-up. While many factors are outside implementers’ control there are steps that donors and their implementers might take to anticipate and potentially ameliorate them. One critical step is to understand policy making, health systems and sociocultural contexts, and being responsive if these change. Hence it is important to build an assessment stage into a grant to help understand and anticipate contextual factors that may influence scale-up, and then to design innovations that respond to these contexts. This implies donors supporting their implementers to do so by building time and resources into the projects they fund.

## Abbreviations

ASHAs: Accredited social health activists
CSOs: Civil society organisations
HEWs: Health extension workers
MDGs: Millennium development goals
MNH: Maternal and newborn health
NRHM: National rural health mission
US: United States.
